# Longitudinal heterogeneity in glioblastoma: moving targets in recurrent versus primary tumors

**DOI:** 10.1186/s12967-019-1846-y

**Published:** 2019-03-20

**Authors:** Niklas Schäfer, Gerrit H. Gielen, Laurèl Rauschenbach, Sied Kebir, Andreas Till, Roman Reinartz, Matthias Simon, Pitt Niehusmann, Christoph Kleinschnitz, Ulrich Herrlinger, Torsten Pietsch, Björn Scheffler, Martin Glas

**Affiliations:** 1 Division of Clinical Neurooncology, Department of Neurology, University Hospital Bonn, 53127 Bonn, Germany; 2 Stem Cell Pathologies, Institute for Reconstructive Neurobiology, University of Bonn, 53127 Bonn, Germany; 3 Institute of Neuropathology, Medical Center Bonn, 53127 Bonn, Germany; 4 Department of Neurosurgery, University Hospital Essen, University Duisburg-Essen, 45147 Essen, Germany; 5 DKFZ Division of Translational Neurooncology at the West German Cancer Center (WTZ), German Cancer Consortium (DKTK), University Hospital Essen, 45147 Essen, Germany; 6 Division of Clinical Neurooncology, Department of Neurology, University Hospital Essen, University Duisburg-Essen, Hufelandstr. 55, 45147 Essen, Germany; 7 Department of Neurosurgery, Medical Center Bonn, 53127 Bonn, Germany; 8 Present Address: Bethel Hospital, 33617 Bielefeld, Germany; 9 Department of Neuro-/Pathology, Oslo University Hospital, Oslo, Norway; 10 Department of Neurology, University Hospital Essen, University Duisburg-Essen, 45147 Essen, Germany

**Keywords:** Glioblastoma, Targeted therapy, Heterogeneity, EGFR, MLPA

## Abstract

**Background:**

Molecularly targeted therapies using receptor inhibitors, small molecules or monoclonal antibodies are routinely applied in oncology. Verification of target expression should be mandatory prior to initiation of therapy, yet, determining the expression status is most challenging in recurrent glioblastoma (GBM) where most patients are not eligible for second-line surgery. Because very little is known on the consistency of expression along the clinical course we here explored common drug targets in paired primary vs. recurrent GBM tissue samples.

**Methods:**

Paired surgical tissue samples were derived from a homogeneously treated cohort of 34 GBM patients. All patients received radiotherapy and temozolomide chemotherapy. Verification of common drug targets included immunohistological analysis of PDGFR-β, FGFR-2, FGFR-3, and mTOR-pathway component (phospho-mTOR^Ser2448^) as well as molecular, MLPA-based analysis of specific copy number aberrations at the gene loci of *ALK*, *PDGFRA*, *VEGFR2/KDR*, *EGFR*, *MET*, and *FGFR1*.

**Results:**

Paired tumor tissue exhibited significant changes of expression in 9 of the 10 investigated druggable targets (90%). Only one target (*FGFR1*) was found “unchanged”, since dissimilar expression was observed in only one of the 34 paired tumor tissue samples. All other targets were variably expressed with an 18–56% discordance rate between primary and recurrent tissue.

**Conclusions:**

The high incidence of dissimilar target expression status in clinical samples from primary vs. recurrent GBM suggests clinically relevant heterogeneity along the course of disease. Molecular target expression, as determined at primary diagnosis, may not necessarily present rational treatment clues for the clinical care of recurrent GBM. Further studies need to analyze the therapeutic impact of longitudinal heterogeneity in GBM.

**Electronic supplementary material:**

The online version of this article (10.1186/s12967-019-1846-y) contains supplementary material, which is available to authorized users.

## Background

In an effort to personalize medicine, target-specific treatment has increasingly been applied in most fields of oncology over the past decade yielding unprecedented benefit on drug response and survival times [1–4]. A broad range of specific drugs has already been established in the field, starting from earliest investigation in clinical trials leading to routine clinical application (Additional file 1: Table S1). Molecularly targeted-therapy is also considered as a rational approach in neurooncology due to the presence of specific molecular alterations that are associated with typical changes in, e.g., glioma-associated signaling pathways [5–8]. One example for successfully targeted therapy in brain tumors is the recent trial of everolimus for treatment of subependymal giant cell astrocytoma in tuberous sclerosis complex (TSC) patients that characteristically have an overactivated mammalian target of rapamycin (mTOR) kinase due to mutations of TSC1/2 genes and subsequent altered activity of the TSC-gene products. Everolimus, an inhibitor of mTOR, has antiproliferative efficacy in these patients [9]. By contrast, literally every targeting effort directed towards the most malignant and most frequent primary brain tumor, i.e. glioblastoma (GBM) has failed in the past [10]. Not a single target-specific compound could be shown to be superior to the already limited efficacy of alkylating chemotherapies [11–17]. The inability of drugs and compounds to cross the blood–brain-barrier and the fact that most of these agents were tested on unselected patient populations, which had not been stratified according to the molecular treatment target (e.g. gene alteration, transmembrane protein etc.) may partly explain these disappointing results. Noteworthy, targeted therapy approaches had been tested to a surprising degree in patient populations suffering from recurrent GBM. This seems particularly challenging, as target gene expression status are not routinely assessed at the time of disease relapse—due to the fact that most patients are not eligible for re-surgery. Clearly, treatment decisions in this setting must be based on the assumption that the target expression status is maintained during the course of disease, that is throughout primary therapy until tumor progression occurs several months later.

The maintenance of a hallmark biomarker, the epigenetic status of O^6^-methyl-guanine-methyltransferase (MGMT) promotor methylation, has respectively been described in primary vs. recurrent GBM tissue [18]. More recent work, however, has begun to highlight shifting genomic/mutational and methylome profiles under the influence of primary treatment schedules in GBM [19, 20], which coincides with the accumulation of discouraging data from clinical trials applying molecular targeted compounds.

Noteworthy, discordant target-/biomarker expression status is frequently observed in other, extraneural solid cancers, particularly upon comparative investigation of primary vs. metastatic disease [21]. This already affects clinical practice in the care of breast and lung cancers where re-biopsy of metastatic lesions is routinely recommended prior to initiation of targeted second-line therapies [22–25].

In an effort to therefore critically assess the applicability of target expression data from primary disease for decisions on second-line treatments at the time of glioblastoma recurrence we here investigated paired surgical tissue samples from a homogeneously treated patient cohort.

## Methods

The retrospective analysis involved patients with histologically confirmed GBM that underwent surgery of primary and recurrent disease at the University Hospital of Bonn in 2003–2014. All patients received similar schemes of first-line treatment, encompassing radiotherapy (60 Gray (Gy) in 30 fractions of 2 Gy to the tumor bed) with concomitant and adjuvant temozolomide-based chemotherapy. Formalin-fixed paraffin-embedded (FFPE) tissue samples were available for all patients, from both, primary and relapse surgery. All samples were classified based on the current revised version of the WHO classification [26]. Stereotactic biopsy material was excluded from this study.

Common drug targets were chosen based on literature support and abundant use in clinical trials in the last decade (see Additional file 1: Tables S1 and S2).

### Tumor tissue samples and immunohistochemistry

FFPE samples were processed for standard H&E staining and immunohistochemical labeling with monoclonal antibodies directed against the platelet-derived growth factor receptor beta (PDGFRβ (2B3); Cell signaling Technology, Inc. New England Biolabs, Frankfurt/Main, Germany, #3175, dilution 1/50), fibroblast growth factor receptor 2 (FGFR2α; R&D Systems Abingdon, UK. #98706, dilution 1/50), fibroblast growth factor receptor 3 (FGFR3 [EPR2305(3)]; Abcam, Cambridge, UK. ab137084, dilution 1/50), and phospho-mTOR^Ser2448^ (49F9; Cell signaling Technology. #2976, dilution 1/50). Phospho-mTOR^Ser2448^ labeling was handled manually as previously described [27]. All other immunohistochemical procedures were performed on a Ventana Benchmark XT Immunostainer (Roche Ventana, Darmstadt, Germany). Two experienced neuropathologists scored the immunohistochemistry reactions visually without access to clinical data. For evaluation of phospho-mTOR^Ser2448^ labeling, scoring specified: negative (0), smaller groups of positive cells (< 50% of total tumor cells; 1), majority of tumor cells positive (> 50% of total tumor cell amount; 2) and (nearly) all tumor cells positive (3). The distribution/density of labeled tumor cells for all other immune reactions were scored semi-quantitatively as negative, low (< 10%), intermediate (10–90%) and high (> 90%).

### Multiplex ligation-dependent probe amplification (MLPA)

DNA from the primary (n = 34) and relapsed (n = 34) tumor samples was isolated using the QIAamp DNA Mini tissue Kit (Qiagen GmbH, Düsseldorf, Germany) according to the manufacturer’s instructions using proteinase K digestion. Careful review of H&E sections ensured a content of vital tumor cells of at least 80% in the respective specimens. Copy number aberrations (CNA) of *ALK, PDGFRA*, *VEGFR2/KDR, EGFR, MET, and FGFR1* were analyzed by MLPA using the SALSA MLPA (MRC Holland, Amsterdam, The Netherlands) P175 A3 (tumor gain probemix) assay according to manufacturer’s instructions [28] and as described previously [29]. After normalization using non-cancer cerebellar tissue (FFPE material), MLPA data were analyzed by Gene Mapper software (Applied Bioscience). A difference of less than threefold standard deviation (SD) from the mean was considered as a lack of CNAs. A difference of plus threefold SD from the mean was considered as a low gain, a value higher or equal 1.5 fold mean as a high gain and a value equal or higher than fivefold mean as a genomic amplification.

## Results

The clinical characteristics of our cohort of 34 GBM patients are presented in Table 1. The median Karnofsky performance status was 90 and for half of the patients, a complete resection was documented at primary surgery as a favorable prognostic factor. Survival times were prolonged, as compared with historical controls: Median progression-free survival was 22.4 months and median overall survival was 35.8 months, indicating that particularly those patients who were eligible for re-resection were included in our cohort.

**Table 1:** Patients’ characteristics

Cohort	n = 34
Age (years)	
Median (range)	61 (22–76)
Gender—n (%)	
Female	14 (41.2)
Male	20 (58.8)
Karnofsky performance score at diagnosis	
Median	90
Extent of primary resection—n (%)	
Complete	17 (50.0)
Partial	6 (17.6)
Open biopsy	11 (32.4)
*IDH*-mutation status—n (%)	
Wildtype	33 (97.1)
Mutated	1 (2.9)
*MGMT* promoter status—n (%)	
Methylated	11 (32.4)
Non-methylated	6 (17.6)
Not determined	17 (50.0)
Progression-free survival	
Median (months)	22.4
Overall survival	
Median (months)	35.8

*IDH* Isocitratdehydrogenase genes 1/2, *MGMT* 0^6^-Methyl-guanine-methyltransferase gene

### Immunohistochemical detection of target expression

**Fig. 1:**
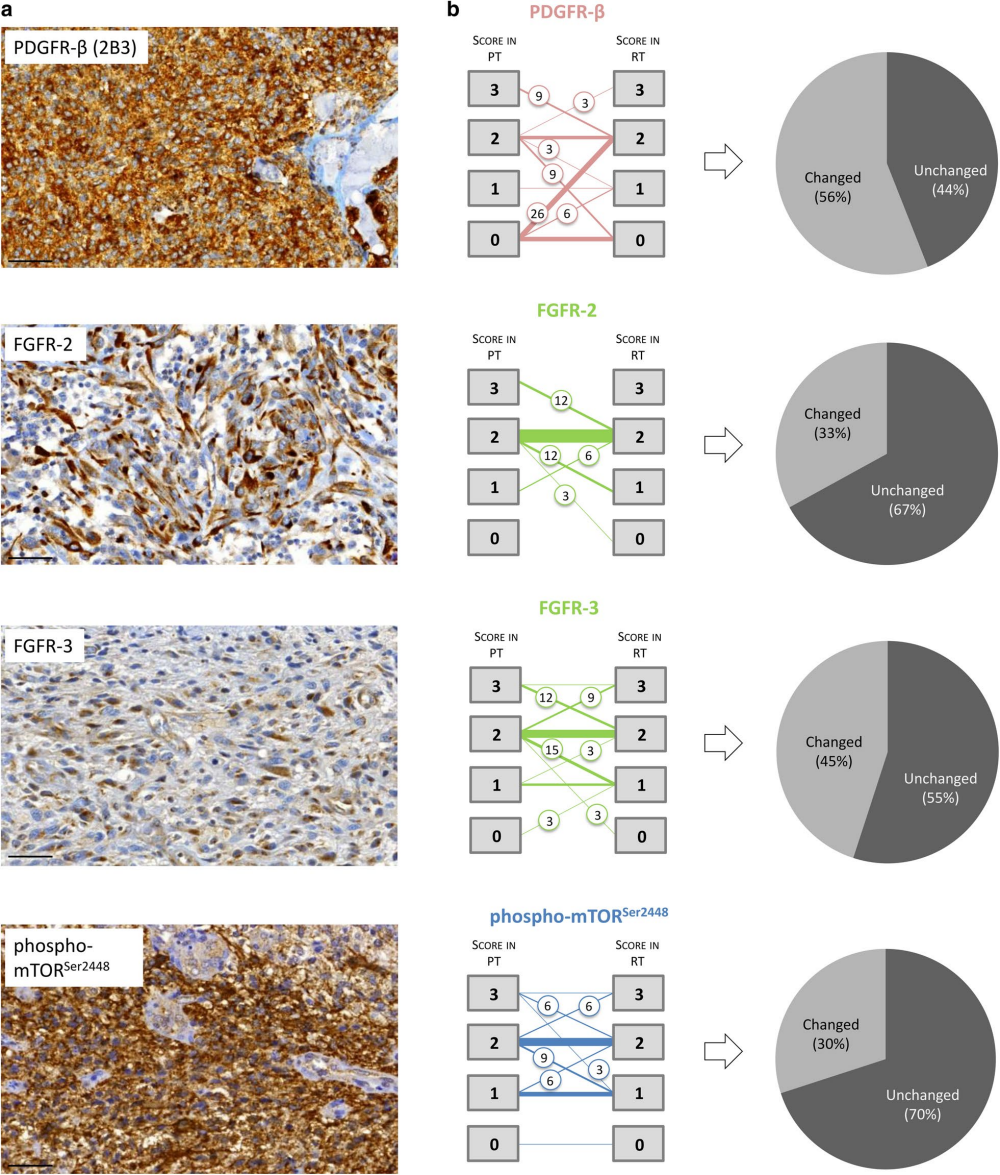
Target investigation by immunohistochemistry.** a** Typical examples of strongly immunoreactive targets (scale bar 50 µm). **b** Shifting target expression as revealed by quantitative scoring (left panel), illustrated by changes per target (right panel). The thickness of lines indicates the number of patients. If the status changed between primary and recurrent tumor tissue, the percentage of affected patients was indicated in circles in the graph (numbers rounded). For PDGFR-β, FGFR-2, and FGFR-3 the numbered boxes represent the portion of positively labeled tumor cells: box 0 = negative; box 1 ≤ 10%; box 2 = 10–90%; box 3 ≥ 90%. Due to spatial/intratumoral inhomogeneous staining results of phosphor-mTOR^Ser2448^: box 0 = negative; box 1 = smaller groups, but < 50% of tumor cells; box 2 = major groups, > 50% of tumor cells; box 3 = nearly all tumor cells positive

Expression of the tyrosine receptor kinase PDGFR-β and its corresponding immunohistochemical scores revealed a high degree of inter-patient heterogeneity in tissue samples from primary and recurrent disease: A cytoplasmic expression pattern of PDGFR-β could be observed in 15/34 (44.1%) of the primary tumors and in 23/34 (67.6%) of the recurrent tumors. No expression in both, primary and recurrent tissue was observed in 8/34 (23.5%) cases. The typical appearance of strong positive immunoreactivity is presented in Fig. 1a. The patient-specific, pair-wise comparison of PDGFR-β revealed in 19/34 (55.9%) tumor samples shifting immunoreactivity scores: An increase of scores in recurrent tumor tissue was detected in 12/34 (35.3%) and a decreased score in 7/34 (20.6%) cases (Fig. 1b). Interestingly, 11/34 (32.4%) of the recurrent tumor samples exposed positive PDGFR-β reactivity, while their paired primary samples were found negative for the target (Fig. 1b, left panel). Altered immunoreactivity scores (independent of extent or direction of change) between the paired primary and recurrent tumor tissues were summarized in Fig. 1b (right panel) and classified as “changed”. In 15/34 (44.1%) of the tumor sample pairs, expression of PDGFR-β was scored as unchanged.

Immunolabeling of FGFR-2, a member of the fibroblast growth factor receptor family, showed a predominantly intermediate or strong expression in the cytoplasm of the tumor cells highlighting the fibrillary processes of the astrocytic differentiated tumor cells. An example of strong immunoreactivity is shown in Fig. 1a. All tissue samples from primary disease showed immunoreactivity for FGFR-2. In the majority of cases, both primary and recurrent samples showed an intermediate expression level (23/34, 67.6%; Fig. 1b). A shifting expression pattern was detected in 11/34 (32.4%) paired samples: In one of these, FGFR-2 expression was lost in the tissue from recurrent disease (1/34, 2.9%). In 8/34 (23.5%) cases, FGFR-2 expression decreased considerably. Increased FGFR-2 expression levels were observed in only 3 cases in tissue from GBM recurrence (3/34, 8.8%) (Fig. 1b, left panel).

FGFR-3, a second member of the fibroblast growth factor family in the panel of investigated GBM targets, revealed a pattern of cytoplasmatic, process-accentuated expression with predominantly intermediate intensity levels (score 2 or higher, see Fig. 1a). 19/34 (55.9%) paired samples exhibited an unchanged expression score (Fig. 1b, right panel). Noteworthy, FGFR-3 expressing primary tumors were also positive in 32/34 (94.1%) of the paired recurrent tumor samples; only 2/34 (5.9%) of the tumor pairs changed their expression profile from negative to positive or from positive to negative, respectively (Fig. 1b, left panel).

Cytoplasmatic expression of the phosphorylated serine/threonine-protein kinase mTOR^Ser2448^ could be detected immunohistochemically in the vast majority of primary as well as recurrent tumor samples (Fig. 1a). Only in one case (2.9%) immunoreactivity with the phospho-mTOR^Ser2448^ antibody could not be revealed in the paired samples at all. Shifting phospho-mTOR^Ser2448^ expression levels could be detected in 10/34 (29.4%) of the paired primary and recurrent tumor samples, while the remaining 24/34 (70.6%) kept a stable expression status at an intermediate intensity (Fig. 1b).

### Copy number variations in glioma-associated target genes

**Fig. 2:**
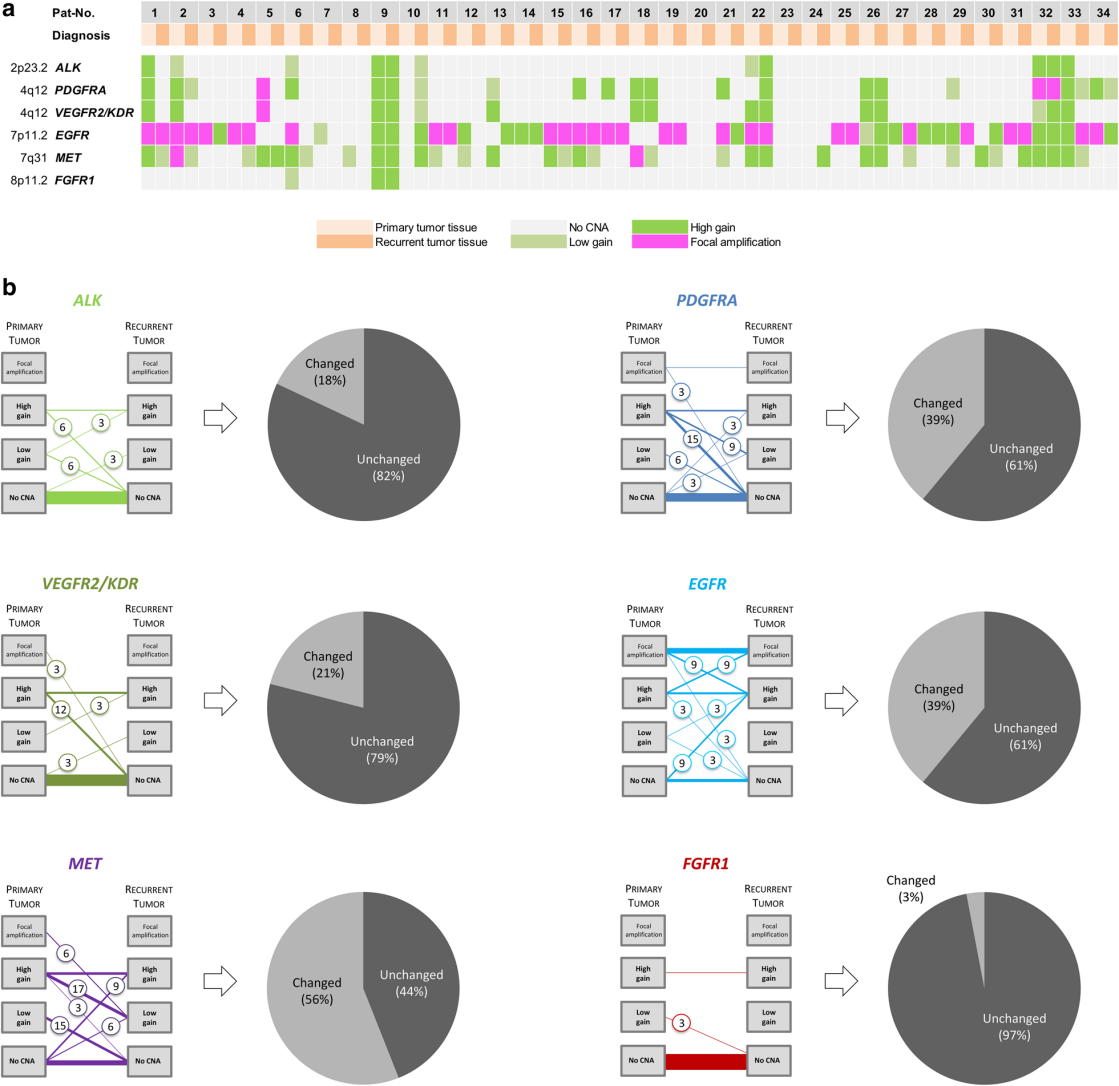
Target investigation by Multiplex ligation-dependent probe amplification (MLPA). **a** Overview of cases and the respective copy number aberrations status of the selected targets. **b** Graphs illustrate correlation of target status in the investigated cohort of paired tissue samples. The thickness of lines visualizes the number of affected patients. A status change between primary and recurrent tumor tissue is indicated by encircled numbers, reflecting the percentage of affected patients (numbers rounded). The boxes indicate the portion of copy number aberrations: No CNA, low gain, high gain, and focal amplification

Pair-wise comparison of CNAs in the glioma-associated genes *ALK*, *PDGFRA*, *VEGFR2/KDR*, *EGFR*, *MET* and *FGFR1* was performed employing MLPA techniques (Fig. 2a). Among the most frequently detected CNAs, *EGFR* amplifications were accounted in 15/34 (44.1%) of the tumor samples from primary disease. Furthermore, 8/34 (23.5%) cases revealed high gains of *EGFR*. The CNA status of *EGFR* remained unchanged in 21/34 (61.8%), but changed in 13/34 (38.2%) of the relapsed tumors. Changes were observed in both directions: In 6/34 (17.6%) cases, CNAs were detected to a lower degree, in 7/34 (20.6%) cases to a higher degree. Newly occurring CNAs in paired tissue from recurring GBM were observed in 3/34 (8.8%) cases and a loss of *EGFR* copy number gains in further 3/34 (8.8%) cases (Fig. 2b).

Less frequently, amplifications affecting *PDGFRA* (2/34; 5.9%), *MET* (2/34; 5.9%) and *VEGFR2/KDR* (1/34; 2.9%) were detected in the tissue from primary disease. However, with the exception of only one case affecting *PDGFRA*, the respective alterations appeared lost at disease recurrence (Fig. 2b).

Although, the majority of tumor samples showed no CNA of *ALK*, *PDGFRA*, *VEGFR2/KDR*, *FGFR1*, and to a lesser extent of *MET* in both primary and recurrent samples (Fig. 2a, b), the concordance of CNA status varied substantially in the subset of cases with alterations. Of these, the highest discordance rate was observed for *PDGFRA* and *MET* with changes in 39% and 56% of cases, respectively (Fig. 2b). There was a tendency for reduction and loss of CNA in the recurrent tumor samples. A lower discordance rate was observed for *ALK*, *VEGFR2/KDR*, and *FGFR1* (changes in 18%, 21%, and 3%; Fig. 2b).

## Discussion

Our comparative analysis of a cohort of 34 paired tissue samples from primary vs. recurrent GBM demonstrates that the distribution and frequency of potentially therapeutic targets can change substantially during the course of disease. The high incidence of dissimilar target expression status in the paired samples suggests a clinically relevant heterogeneity that may additionally be affected by the effect of primary therapy, including radio-/chemotherapy with temozolomide. Thus, the molecular target expression status, as determined at the time of primary resection, may not necessarily present rational treatment clues for the care of recurrent GBM that occurs 6–9 months later [10]. This has immediate implications for clinical practice, as current routine procedures rely on the results from analysis of primary glioblastoma resection for the prediction of personalized second-line therapy. Our data show that this practice carries a non-neglectable risk and should be reconsidered.

GBM recurrence occurs inevitably in almost every patient and standards of care for a meaningful second-line treatment approach have not been established yet [30]. Molecularly targeted therapy remains a favorable concept in personalized medicine that has led to considerably increased survival times in many other cancers in the past. Our data imply that prior to starting targeted salvage therapy in recurrent GBM, at least two issues need to be addressed. First, verification of target expression in the recurrent tumor tissue should be mandatory to precisely address the original idea of molecularly targeted therapy. This underlines the need of a re-biopsy and tissue analysis at time of disease recurrence, as already recommended by others [19], however, the risk of an additional neurosurgical procedure should be carefully considered on a case-to-case basis. Second, diagnostic standards should be easy to implement in cancer centers where standardized genomic and immunohistochemistry procedures would enable expedited treatment initiation.

Re-evaluation of target expression status in recurrent tumor tissue would have the greatest impact if that target is known to be expressed in primary tissue but, due to a variety of possible reasons, lost in recurrent disease and vice versa. In that case, putatively eligible treatment choices could be disregarded due to the absence of respective targets in the primary tumor tissue or, a chosen molecularly targeted therapy could lack efficacy due to the absence of respective targets at tumor relapse. Stressing the example of a PDGFR-β-directed treatment approach, data from our cohort analysis would imply that 35% of eligible patients would probably be provided with an incorrect treatment decision, unless the evaluation of target expression status would be conducted from tissue at primary and recurrent disease.

Limitations to this assumption come from the yet uncharacterized correlation of target expression status at recurrence of disease, the absence of clearly defined cut-off values for positively scored targets, and from the unclear maximum extent of treatment responses under molecularly target therapy. In addition, the predictive value for many of the candidate targets is not well established in recurrent GBM, and it is not sufficiently investigated whether the observation of shifting target expression status is functionally relevant for the further progression of disease. In addition to the promising results with targeting alterations in the mTOR pathway [9, 31], for most signaling pathways, appropriate diagnostic tools remain to be established. And, ultimately, the tremendous extent of heterogeneity in GBM will continue to pose challenges to the successful conversion of data from investigation of recurrent tumor tissue towards clinical practice. There is a well-documented and considerable extent of inter-patient heterogeneity [5, 32, 33], and there is an increasingly recognized intra-patient heterogeneity that becomes evident, e.g., by the dynamic expansion of coexisting tumor subclones under therapy, which needs to be considered as molecularly distinct [34–38].

Moreover, we cannot exclude that epigenetic variations resemble an alternative mechanism for the variation of target expression. Beside this, gene expression networks, cell lineage and phenotype of individual cells are emerging contributors to the complexity and intra-tumoral heterogeneity of glioblastoma and should be subject of further detailed studies.

Our study furthermore highlights the need to carefully implement, in future diagnostic schemes, the investigation of longitudinal heterogeneity that may be therapy-driven along the course of disease [39]. Future diagnostics may not rely on a single biopsy for sufficient characterization of a tumor’s molecular profile, and thus, clinical standards need to be developed that enable safe acquisition of diagnostic biomaterial from every GBM patient, even those that are not eligible for major re-surgery measures.

Taken together, the molecular characteristics of recurrent tumors cannot sufficiently be predicted from analysis of primarily resected GBM tissue. New trial concepts should consider re-surgery or re-biopsy to enable tissue analysis along the clinical course in GBM to assess the efficacy prior to initiation of a molecularly targeted therapy. In this context, so called “I-Spy”-like trial designs [40], that treat patients depending on their individual molecular profile for a variety of preselected targets with the corresponding compounds could be developed into an appropriate future scenario.

## Conclusions

We could demonstrate a high incidence of dissimilar target expression status in clinical samples from primary vs. recurrent GBM. Molecular target expression, as determined at primary diagnosis, may not necessarily present rational treatment clues for the clinical care of recurrent GBM. Thus, second-line therapy require verification of target expression status and further studies need to analyze the therapeutic impact of these findings.

## Additional file

**Additional file 1: Table S1.** List of target-directed compounds that are either approved in the United States by the Federal Drug Administration (FDA) or under investigation in clinical trials. **Table S2.** Overview of compounds directed against selected targets.

## Abbreviations

CNA: copy number aberration
DNA: desoxyribunucleic acid
FFPE: formalin-fixed paraffin-embedded
GBM: glioblastoma
Gy: gray
IDH: isocitratdehydrogenase gene
MGMT: O^6^-methyl-guanine-methyltransferase gene
MLPA: multiplex ligation-dependent probe amplification
mTOR: mammalian target of rapamycin
SD: standard deviation
TSC: tuberous sclerosis complex
