# Supplementary material for: Longitudinal heterogeneity in glioblastoma: moving targets in recurrent versus primary tumors
Source: J Transl Med. 2019 Mar 20;17:96. doi: 10.1186/s12967-019-1846-y (PMC6425567; doi:10.1186/s12967-019-1846-y)
Supplement: Supplementary file 1 — Additional file 1: Table S1. List of target-directed compounds that are either approved in the United States by the Federal Drug Administration (FDA) or under investigation in clinical trials. Table S2. Overview of compounds directed against selected targets. [file 12967_2019_1846_MOESM1_ESM.docx]

**Appendix**

**Selection of potential targets**

PDGFR-α, PDGFR-β, and EGFR represent typical elements of molecular changes in malignant glioma, whereas FGFR and MET are more unfrequent genes that exhibit mutations or amplifications.^5,6,41^ Receptors of VEGF are strongly connected with angiogenesis (for review see Plate (1999)),^42^ which plays an essential role in tumor biology. With respect to clinical data we selected a mTOR-pathway component and ALK, since GBM patients with a non-methylated MGMT promoter and phosphorylated mTOR^Ser2448^ may benefit from treatment with temsirolimus^31^ Furthermore, ALK-directed treatment in lung cancer patients is very effective and ALK may also represent a target in other cancer types^43,44^ The vast availability of compounds directed against the selected targets is shown in additional file table 2.

| **Compound** | **Target** | **Cancer disease** | **FDA Status** | **NCT identifier** | **Reference** |
| --- | --- | --- | --- | --- | --- |
| Afatinib | EGFR, ErbB2 | Head and neck cancer | Phase III | NCT01345682 | Machiels et al, Lancet Oncol, 2015 |
|  |  | Lung cancer | Approved | NCT01523587 | Soria et al, Lancet Oncol, 2015 |
| Alectinib | ALK | Lung cancer | Approved | NCT01588028 | Gadgeel et al, Lancet Oncol, 2014 |
| Alisertib | Aurora A | Breast cancer | Phase II | NCT01045421 | Melichar et al, Lancet Oncol, 2015 |
|  |  | Lung cancer | Phase II | NCT01045421 | Melichar et al, Lancet Oncol, 2015 |
|  |  | Lymphoma | Phase III | NCT01466881 | Barr et al, J Clin Oncol, 2015 |
| Alvocidib | Cdk9/1/2/4/6 | Leukemia | Orphan drug | NCT00098371 | Lin et al, J Clin Oncol, 2009 |
| AT9283 | Aurora A/B, Abl-1, Jak2 | Solid tumors | Phase II | NCT00985868 | Moreno et al, Clin Cancer Res, 2015 |
| Axitinib | VEGFR1-3, c-Kit, PDGFRβ | Kidney cancer | Approved | NCT00678392 | Rini et al, Lancet, 2011 |
| Barasertib | Aurora B | Leukemia | Phase III | NCT00952588 | Loewenberg et al, Blood, 2011 |
| Bicalutamide | Androgen receptor | Breast cancer | Phase II | NCT00468715 | Gucalp et al, Clin Cancer Res, 2013 |
| Bosutinib | Abl-1, Src | Breast cancer | Phase II | NCT00319254 | Campone et al, Ann Oncol, 2012 |
|  |  | Leukemia | Approved | NCT00261846 | Khoury et al, Blood, 2012 |
| Cabozantinib | c-Met, c-Kit, RET, VEGFR2 | Kidney cancer | Approved | NCT01865747 | Choueiri et al, N Engl J Med, 2015 |
|  |  | Thyroid cancer | Approved | NCT00704730 | Elisei et al, J Clin Oncol, 2013 |
| Cediranib | VEGFR2 | Ovarian cancer | Phase III | NCT00532194 | Ledermann et al, Lancet, 2016 |
|  |  | Sarcoma | Phase II | NCT00942877 | Kummar et al, J Clin Oncol, 2013 |
|  |  | Cervical cancer | Phase II | NCT01229930 | Sydmonds et al, Lancet Oncol, 2015 |
| Ceritinib | ALK | Lung cancer | Approved | NCT01283516 | Shaw et al, N Engl J Med, 2014 |
| Cetuximab | EGFR | Colorectal cancer | Approved | NCT00079066 | Jonker et al, N Engl J Med, 2007 |
| Crizotinib | ALK, c-Met | Lung cancer | Approved | NCT01154140 | Solomon et al, N Engl J Med, 2014 |
|  |  | Lymphoma | Phase II | NCT00939770 | Mossé et al, Lancet Oncol, 2013 |
| Dabrafenib | B-Raf | Melanoma | Approved | NCT01227889 | Hauschild et al, Lancet, 2012 |
| Dasatinib | Abl-1 | Leukemia | Approved | NCT00481247 | Kantarjian et al, N Engl J Med, 2010 |
| Enzalutamide | Androgen receptor | Prostate cancer | Approved | NCT00974311 | Scher et al, N Engl J Med, 2012 |
| Erlotinib | EGFR | Lung cancer | Approved | NCT00373425 | Shepherd et al, N Engl J Med, 2005 |
| Everolimus | mTOR | Breast cancer | Approved | NCT00863655 | Baselga et al, N Engl J Med, 2012 |
|  |  | Endometrial cancer | Phase II | NCT01068249 | Slomovitz et al, J Clin Oncol, 2015 |
|  |  | Gastric cancer | Phase III | NCT00519324 | Doi et al, J Clin Oncol, 2010 |
|  |  | Kidney cancer | Approved | NCT00410124 | Motzer et al, Lancet, 2008 |
|  |  | Lymphoma | Phase III | NCT00516412 | Renner et al, Haematologica, 2012 |
|  |  | Neuroendocrine tumor | Approved | NCT01524783 | Yao et al, Lancet, 2016 |
|  |  | Urothelial cancer | Phase II | NCT00714025 | Seront et al, Ann Oncol, 2012 |
| Flutamide | Androgen receptor | Prostate cancer | Approved | NCT02175212 | Zapatero et al, Lancet Oncol, 2015 |
| Foretinib | c-Met, VEGFR2 | Kidney cancer | Phase II | NCT00726323 | Choueiri et al, J Clin Oncol, 2013 |
| FP-1039 | FGF | Solid tumors | Phase II | NCT00687505 | Tolcher et al, Ann Oncol, 2016 |
| Gefitinib | EGFR | Lung cancer | Approved | NCT00322452 | Mok et al, N Engl J Med, 2009 |
| Idasanutlin | Mdm2 | Leukemia | Phase III | NCT01773408 | Reis et al, Haematologica, 2016 |
| Imatinib | Abl-1, c-Kit, PDGFRα/β, RET | Chordoma | Phase II | NCT00150072 | Stacchiotti et al, J Clin Oncol, 2012 |
|  |  | Gastrointestinal stroma tumor | Approved | NCT00009906 | Verweij et al, Lancet, 2004 |
|  |  | Leukemia | Approved | NCT00006343 | Druker et al, N Engl J Med, 2006 |
|  |  | Melanoma | Phase II | NCT00881049 | Guo et al, J Clin Oncol, 2011 |
| Lapatinib | EGFR, ErbB2 | Breast cancer | Approved | NCT00078572 | Geyer et al, N Engl J Med, 2006 |
|  |  | Kidney cancer | Phase III | n/a | Ravaud et al, J Clin Oncol, 2008 |
| Lenvatinib | FGFR1-4, VEGFR1-3, PDGFRα, c-Kit, RET | Hepatocellular carcinoma | Phase III | n/a | Ikeda et al, Clin Cancer Res, 2016 |
|  |  | Kidney cancer | Phase II | NCT01136733 | Motzer et al, Lancet Oncol, 2015 |
|  |  | Thyroid cancer | Approved | NCT01321554 | Schlumberger et al, N Engl J Med, 2015 |
| Linifanib | c-Kit, VEGFR1-3, PDGFRβ, CSF1R | Lung cancer | Phase II | NCT00716534 | Ramalingam et al, J Clin Oncol, 2015 |
|  |  | Kidney cancer | Phase II | NCT00486538 | Tannir et al, Eur J Cancer, 2011 |
| Necitumumab | EGFR | Lung cancer | Approved | NCT00981058 | Thatcher et al, Lancet Oncol, 2015 |
| Neratinib | EGFR, ErbB2 | Breast cancer | Phase III | NCT00300781 | Burstein et al, J Clin Oncol, 2010 |
|  |  | Lung cancer | Phase II | NCT00266877 | Sequist et al, J Clin Oncol, 2010 |
| Nilotinib | Abl-1, c-Kit | Leukemia | Approved | NCT00471497 | Saglio et al, N Engl J Med, 2010 |
| Nilutamide | Androgen receptor | Prostate cancer | Approved | n/a | Dijkman et al, J Urol, 1997 |
| Nintedanib | FGFR1-3, VEGFR1-3, PDGFRα/β | Colorectal cancer | Phase III | NCT00904839 | Van Cutsem et al, Ann Oncol, 2015 |
|  |  | Lung cancer | Phase III | NCT00805194 | Reck et al, Lancet Oncol, 2014 |
|  |  | Ovarian cancer | Phase III | NCT01015118 | Du Bois et al, Lancet Oncol, 2016 |
| Osimertinib | EGFR | Lung cancer | Approved | NCT01802632 | Jänne et al, N Engl J Med, 2015 |
| Palbociclib | Cdk4/6 | Breast cancer | Approved | NCT01942135 | Turner et al, N Engl J Med, 2015 |
|  |  | Lymphoma | Phase II | NCT00420056 | Leonard et al, Blood, 2012 |
|  |  | Sarcoma | Phase II | NCT01209598 | Dickson et al, J Clin Oncol, 2013 |
| Panitumumab | EGFR | Colorectal cancer | Approved | NCT00364013 | Douillard et al, N Engl J Med, 2013 |
| Pazopanib | FGFR3, c-Kit, PDGFRα/β, VEGFR1-3 | Cervical cancer | Phase II | NCT00430781 | Monk et al, J Clin Oncol, 2010 |
|  |  | Kidney cancer | Approved | NCT00720941 | Motzer et al, N Engl J Med, 2013 |
|  |  | Lung cancer | Phase III | NCT00367679 | Altorki et al, J Clin Oncol, 2010 |
|  |  | Neuroendocrine tumor | Phase II | NCT00454363 | Phan et al, Lancet Oncol, 2015 |
|  |  | Sarcoma | Approved | NCT00753688 | van der Graaf et al, Lancet, 2012 |
|  |  | Thyroid cancer | Phase II | NCT00625846 | Bible et al, Lancet Oncol, 2010 |
| Pertuzumab | ErbB2 | Breast cancer | Approved | NCT00567190 | Swain et al, N Engl J Med, 2015 |
| Ponatinib | Abl-1, FGFR1-3, c-Kit, PDGFRα, RET, VEGFR2 | Leukemia | Approved | NCT01207440 | Cortes et al, N Engl J Med, 2013 |
| Pralatrexate | DHFR | Lymphoma | Approved | NCT00364923 | O'Connor et al, J Clin Oncol, 2011 |
| RG7112 | Mdm2 | Leukemia | Phase I | NCT00623870 | Andreeff et al, Clin Cancer Res, 2016 |
| Ramucirumab | VEGFR2 | Colorectal cancer | Phase III | NCT01183780 | Tabernero et al, Lancet Oncol, 2015 |
|  |  | Gastric cancer | Approved | NCT00917384 | Fuchs et al, Lancet, 2014 |
|  |  | Lung cancer | Approved | NCT01168973 | Garon et al, Lancet, 2014 |
| Regorafenib | Abl-1, c-Kit, FGFR1/2, PDGFRα/β, B-Raf, VEGFR1-3 | Colorectal cancer | Approved | NCT01584830 | Li et al, Lancet Oncol, 2015 |
|  |  | Gastrointestinal strom tumor | Approved | NCT01271712 | Demetri et al, Lancet, 2013 |
|  |  | Hepatocellular carcinoma | Phase III | NCT01003015 | Bruix et al, Eur J Cancer, 2013 |
|  |  | Kidney cancer | Phase II | NCT00664326 | Eisen et al, Lancet Oncol, 2012 |
| Sonidegib | Smo | Skin cancer | Approved | NCT01327053 | Migden, Lancet Oncol, 2015 |
| Sorafenib | B-Raf, C-Raf, FGFR1, c-Kit, PDGFRβ, RET, VEGFR1-3 | Breast cancer | Phase III | NCT01234337 | Baselga et al, J Clin Oncol, 2012 |
|  |  | Hepatocellular carcinoma | Approved | NCT00105443 | Llovet et al, N Engl J Med, 2008 |
|  |  | Kidney cancer | Approved | NCT00073307 | Escudier et al, N Engl J Med, 2007 |
|  |  | Lung cancer | Phase III | NCT00600015 | Spigel et al, J Clin Oncol, 2011 |
|  |  | Thyroid cancer | Approved | NCT00984282 | Brose et al, Lancet, 2014 |
| Sunitinib | c-Kit, VEGFR1-3, PDGFRβ, RET | Gastrointestinal strom tumor | Approved | NCT00075218 | Demetri et al, Lancet, 2006 |
|  |  | Kidney cancer | Approved | NCT00098657 | Motzer et al, N Engl J Med, 2007 |
|  |  | Lung cancer | Phase III | NCT00453154 | Ready et al, J Clin Oncol, 2015 |
|  |  | Neuroendocrine tumor | Approved | NCT00428597 | Raymond et al, N Engl J Med, 2011 |
|  |  | Sarcoma | Phase II | NCT00474994 | George et al, J Clin Oncol, 2009 |
| T-DM1 | ErbB2 | Breast cancer | Approved | NCT00829166 | Verma et al, N Engl J Med, 2012 |
| Temsirolimus | mTOR | Breast cancer | Phase II | n/a | Chan et al, J Clin Oncol, 2005 |
|  |  | Endometrial cancer | Phase II | NCT00072176 | Oza et al, J Clin Oncol, 2011 |
|  |  | Kidney cancer | Approved | NCT00065468 | Hudes et al, N Engl J Med, 2007 |
|  |  | Lymphoma | Phase III | NCT00117598 | Hess et al, J Clin Oncol, 2009 |
|  |  | Neuroendocrine tumor | Phase II | NCT01010126 | Hobday et al, J Clin Oncol, 2015 |
|  |  | Sarcoma | Phase III | NCT01016015 | Schwartz et al, Lancet Oncol, 2013 |
| Tivantinib | c-Met | Hepatocellular carcinoma | Phase III | NCT00988741 | Santoro et al, Lancet Oncol, 2013 |
| Trastuzumab | ErbB2 | Breast cancer | Approved | NCT00021255 | Slamon et al, N Engl J Med, 2011 |
|  |  | Gastric cancer | Approved | NCT01041404 | Bang et al, Lancet, 2010 |
| Vandetanib | EGFR, RET, VEGFR2 | Lung cancer | Phase III | NCT00312377 | Herbst et al, Lancet Oncol, 2010 |
|  |  | Thyroid cancer | Approved | NCT00537095 | Leboulleux et al, Lancet Oncol, 2012 |
| Vatalanib | VEGFR1-3, c-Kit, PDGFRβ | Colorectal cancer | Phase III | NCT00056446 | Van Cutsem et al, J Clin Oncol, 2011 |
|  |  | Gastrointestinal strom tumor | Phase II | NCT00117299 | Joensuu et al, Ann Oncol, 2008 |
|  |  | Lung cancer | Phase II | NCT00160043 | Gauler et al, Ann Oncol, 2012 |
|  |  | Pancreatic cancer | Phase II | NCT00226005 | Dragovich et al, Cancer Chemother Pharmacol, 2014 |
| Vemurafenib | B-Raf | Leukemia | Phase II | NCT01524978 | Hyman et al, N Engl J Med, 2015 |
|  |  | Leukemia | Phase II | NCT01711632 | Tiacci et al, N Engl J Med, 2015 |
|  |  | Melanoma | Approved | NCT01006980 | McArthur et al, Lancet Oncol, 2014 |
| Vismodegib | Smo | Skin cancer | Approved | NCT00833417 | Sekulic et al, N Engl J Med, 2012 |

**Table S1** List of target-directed compounds that are either approved in the United States by the Federal Drug Administration (FDA) or under investigation in clinical trials

| Potential target | Compounds directed against target |
| --- | --- |
| PDGFR-α | Amuvatinib, Imatinib, Lenvatinib, Nintedanib, Pazopanib, Ponatinib, Regorafenib, Sunitinib, XL820 |
| PDGFR-β | Axitinib, Dasatinib, Dovitinib, Imatinib,  Linifanib, Nintedanib, Pazopanib, Regorafenib, Sorafenib, Sunitinib, Vatalanib, XL820, XL999 |
| FGFR-1 | Dovitinib, Lenvatinib, Nintedanib, Ponatinib, Regorafenib, Sorafenib, SU4984, XL228, XL999 |
| FGFR-2 | Dovitinib, Lenvatinib, Nintedanib, Ponatinib, Regorafenib, SU4984, XL228 |
| FGFR-3 | Dovitinib, Lenvatinib, Nintedanib, Pazopanib, Ponatinib, XL228, XL999 |
| EGFR | Afatinib, Brigatinib, Canertinib, Cetuximab, Erlotinib, Gefitinib, IGN311, INSM-18, Lapatinib, Matuzumab, Necitumumab, Neratinib, Osimertinib, Panitumumab, Trastuzumab, Vandetanib, Zalutumumab |
| MET | AMG458, Amuvatinib, BMS777607,  Cabozantinib, Capmatinib, Crizotinib,  Foretinib, Golvatinib, JNJ38877605,  MK2461, Onartuzumab, PF04217903,  PHA665752, SU11274, Tivantinib |
| VEGFR-2/KDR | Axitinib, Cabozantinib, Cediranib, CYC116, Dovitinib, Foretinib, Golvatinib, IMC1C11, Lenvatinib, Linifanib, MK2461, Nintedanib, Pazopanib, Ponatinib, RAF265, Ramucirumab, Regorafenib, Sorafenib, Sunitinib, Vandetanib, Vatalanib, XL820, XL999 |
| VEGFR-3 | Axitinib, Dovitinib, IMC1C11, Lenvatinib,  Linifanib, Nintedanib, Pazopanib, Regorafenib, Sorafenib, Sunitinib, Vatalanib |
| ALK | Alectinib, Brigatinib, CEP-37440, Ceritinib, Crizotinib, Entrectinib, Lorlatinib, TSR-011, X-396 |
| mTOR | Temsirolimus, Voxtalisib |

**Table S2** Overview of compounds directed against selected targets

**Additional file references**

41) Puputti M, Tynninen O, Sihto H et al. Amplification of KIT, PDGFRA, VEGFR2, and EGFR in gliomas. Mol Cancer Res 2006; 4(12): 927–934.

42) Plate KH. Mechanisms of angiogenesis in the brain. J Neuropathol Exp Neurol 1999; 58(4): 313–320.

43) Solomon BJ, Mok T, Kim DW et al. First-line crizotinib versus chemotherapy in ALK-positive lung cancer. N Engl J Med 2014; 371(23): 2167–21­­77.

44) Wellstein A. ALK receptor activation, ligands and therapeutic targeting in glioblastoma and in other cancers. Front Oncol 2012; 2: 192.
